# The independent effects of second hand smoke exposure and maternal body mass index on the anthropometric measurements of the newborn

**DOI:** 10.1186/1471-2458-13-1058

**Published:** 2013-11-09

**Authors:** Hayfaa A Wahabi, Ahmed A Mandil, Rasmieh A Alzeidan, Ahmed A Bahnassy, Amel A Fayed

**Affiliations:** 1 Sheikh Bahamdan Research Chair of Evidence-based Healthcare and Knowledge translation, College of Medicine, King Saud University, P.O Box 102799, Riyadh 11685, Kingdom of Saudi Arabia; 2 Department of Family and Community Medicine, College of Medicine, King Saud University, Riyadh, Kingdom of Saudi Arabia; 3 Department of Family and Community Medicine, College of Medicine, King Fahad Medical City, King Saud Bin Abdulaziz University for Health Sciences, Riyadh, Kingdom of Saudi Arabia; 4 College of Health Sciences, King Saud Ben AbdulAziz University, Riyadh, Kingdom of Saudi Arabia

**Keywords:** Secondhand smoke, Maternal body mass index, Newborn anthropometry, Saudi Arabia

## Abstract

**Background:**

Exposure to tobacco smoke during pregnancy, whether as active smoking or by exposure to secondhand smoke (SHS), is associated with adverse pregnancy outcomes including low birth weight (LBW) and small for gestational age infants due to the effect of tobacco on the anthropometric measurements of the newborn. This effect might be masked by maternal obesity as it increases fetal weight. The objectives of this study were to estimate the independent effects of maternal exposure to SHS and maternal body mass index (BMI) on the anthropometric measurements and on the prevalence of macrosomia and LBW among term infants.

**Methods:**

Data were collected from women in the postnatal ward following delivery. Participants were stratified into six groups based on the BMI (underweight <18 kg/m^2^, non-obese 18–29.9 kg/m^2^, and obese ≥30 kg/m^2^) and the SHS exposure status (exposed and non- exposed), to examine the independent effects of BMI and SHS on infants’ anthropometry. Multiple regression analysis was used to explore the independent associations between the six groups and the risk of delivering a macrosomic or LBW infant.

**Results:**

Infants of women exposed to SHS had significantly reduced anthropometric measurements compared to infants of unexposed women. The odds of delivering a macrosomic baby increased to 9-fold for women with BMI of ≥30 kg/m^2^ compared to non-obese women; odds ratio (OR) 9.18, 95% Confidence Interval (CI) (1.01, 9.37); p = 0.04, this risk was attenuated to 1.5-fold in women exposed to SHS, OR 1.53, 95% CI (1.19, 12.1); p < 0.0001. The odds of delivering an LBW infant were more than doubled in underweight women compared to non-obese women, OR 2.15, 95% CI (1.001, 4.57); p = 0.034, and were further increased to almost 3-fold for women who were exposed to SHS, OR 2.71, 95% CI (1.82,4.045); p = 0.02.

**Conclusion:**

Exposure to SHS was associated with reduced anthropometric measurements of the newborn and increased rate of LBW infants, irrespective of maternal BMI. Maternal obesity was associated with increased risk of delivering a macrosomic infant; conversely maternal underweight was associated with increased risk of delivering an LBW infant.

## Background

Exposure to tobacco smoke during pregnancy, whether as active smoking or by exposure to secondhand smoke (SHS), is associated with adverse pregnancy outcomes [1-3]. A well-recognized and documented adverse effect of maternal tobacco exposure is that on birth weight and anthropometric measurements of the newborn leading to high prevalence of low birth weight (LBW) and small for gestational age infants [3,4]. This effect might be masked by maternal obesity, which operates in the opposite direction by increasing fetal weight and anthropometric measurements [5]. However infants of normal birth weight, who have been exposed to tobacco smoke, still suffer the adverse effect of exposure as demonstrated by an earlier study which showed that the mortality curve of infants exposed to smoking at any measure of birth weight was higher than for unexposed infants [6].

Previous studies on the combined effect of in-utero exposure to tobacco smoke and maternal body mass index (BMI) showed that; both underweight and obese women and their infants, who were exposed to tobacco smoke during pregnancy, suffered increased adverse pregnancy outcomes compared to the same category of BMI of non-exposed mothers [7,8].

The few published reports about tobacco use and SHS exposure during pregnancy in the Arab World are limited by either the small number of participants [9], or the difference in culture and social norms between geographical areas which limit generalization of results [10]. The only published study from Saudi Arabia regarding prevalence of tobacco use in pregnancy, showed that despite the low prevalence of active smoking among Saudi women, more than 30% of Saudi pregnant women were exposed to SHS, with evidence of adverse effects on the newborn’s weight and head circumference [3].

A recent study estimated the prevalence of obesity and overweight among Saudi pregnant women to be more than 52%, with evident increased rate of obstetric complications, birth weight and prevalence of macrosomia among the infants of obese and overweight mothers compared to those of normal weight [11].

The objectives of this study were to estimate the independent effects of maternal exposure to SHS and maternal BMI on the anthropometric measurements of term infants and on the prevalence of macrosomia and LBW.

## Methods

This study was conducted at the postnatal ward of King Khalid University Hospital (KKUH) in the Kingdom of Saudi Arabia for the period of 12 months from the 1st of October 2011 to 30th of September 2012. KKUH is a tertiary referral center; includes a neonatal intensive care unit (NICU) and in vitro fertilization unit. The obstetrics department provides services for 3500–4000 deliveries per year.

The study was designed to investigate the independent effects of maternal BMI and maternal exposure to SHS on the new-borns’ anthropometric measurements (birth weight, length, and head circumference), LBW < 2.5 kg and macrosomia (≥4.0 kg) in term infants.

Consecutive women who consented to join the study and met the inclusion criteria were enrolled. The inclusion criteria were:

1. Women with singleton pregnancy.

2. Term delivery (≥ 37 gestational weeks counted from the last menstrual period and/or early ultra-sound scan).

3. Women who did not smoke during the index pregnancy and were exposed to SHS.

4. Women who did not smoke during the index pregnancy and were not exposed to SHS.

We excluded from this study women with unknown smoking status and those who did not have recorded weight and/or height at time of booking to the antenatal clinic.

Data were collected using a predesigned data collection sheet from women in the postnatal ward within 24–48 hours following delivery, by nurses and researchers who were trained to collect the data. Demographic characteristics and pregnancy outcomes data were extracted from the medical records, while tobacco use data were obtained by interview. The researchers who conducted the interviews were not aware of the data extracted from the medical records.

Women who met the inclusion criteria and consented to the study were asked about their exposure to SHS, which was defined as occurring when a woman, who did not smoke at all whilst pregnant, lived with a household member (husband, son, daughter or other relatives) who was reported to smoke during the index pregnancy. We did not assess occupational exposure. Duration of exposure to SHS was not reported in this study as only 30% of the participants could recall the duration of exposure.

Women were booked for their first antenatal visit during the first or the second trimester of pregnancy, subject to availability of appointments.

The data collected from the antenatal records included; maternal age, parity, maternal height and weight recorded during the first antenatal visit, from which, BMI was calculated according to the following equation; BMI = weight (kg)/height (m)^2^[12], in addition to any antenatal events including the occurrence of preeclampsia and pre-existed or gestational diabetes (GDM) as per antenatal record diagnosis. These variables were extracted and analysed as confounders due to their known influence on newborn anthropometry.

Data of the newborn included gestational age at delivery, weight, length and head circumference.

To investigate the independent effects of maternal obesity and exposure to SHS on the newborn’s anthropometric measurements, women were stratified into three groups based on the BMI; underweight (BMI <18 kg/m^2^) non-obese (BMI 18–29.99 kg/m^2^) and obese (BMI ≥ 30 kg/m^2^). Further stratification of the study population into a total of six groups was based on the results of the SHS exposure; where non- obese women who reported non-exposure to SHS were considered the control group. We compared the birth weight, length and head circumference, of infants of mothers who were exposed to SHS to those of mothers who were exposed, stratified according to the BMI. In addition we estimated the risk of delivering a macrosomic or an LBW baby by calculating the odds ratio (OR) for each category of women.

### Data analysis

Data were analysed using Statistical Package for the Social Sciences (SPSS), Version 20 (SPSS Inc., Chicago, IL, USA). We compared means using either the Student’s t test or analysis of variance (ANOVA) for continuous variables, as appropriate, and Pearson's chi-squared test for categorical variables; non-parametric tests were used as appropriate. Univariate analysis was performed to compare the anthropometric measurements of infants of women who were exposed to SHS to those of women who were not exposed and to evaluate the associations between maternal BMI and exposure to SHS with anthropometric measurements.

Stepwise multiple logistic regression analysis was used to explore the independent associations between the six groups regarding the risk of delivering a macrosomic or an LBW infant, considering non-obese women who reported non-exposure to SHS as the reference group. Adjusted ORs were calculated and the following variables were adjusted for in the regression model; maternal age, parity, gestation age 37–42 weeks, GDM, preeclampsia and pre-existing diabetes mellitus. P value of < 0.05 was considered significant.

Ethical approval was sought and granted before commencing the study from the institutional review board of College of Medicine King Saud University.

## Results

During the study period there were 3766 deliveries of them 3 women self-reported active smoking. A total of 3231 women met the inclusion criteria and consented to the study, of those 798 (24.7%) self-reported exposure to SHS while 2433 (75.3%) did not report such exposure. Of the study population 1404 (43.5%) were obese (BMI ≥ 30 kg/m^2^) while 21 (0.65%) were under weight (BMI < 18 kg/m^2^). The demographic characteristics of the women exposed and not exposed to SHS are shown in Table 1. Women exposed to SHS, were younger and of lower parity yet they had similar frequency of pregnancy complications and risk factors known to affect the pregnancy outcomes (Table 1).

**Table 1:** Maternal and infant characteristics, of women exposed and non-exposed to SHS

**Variable**	**Women non-exposed to SHS (N = 2433)**	**Women exposed to SHS (N = 798)**	**P value**
**Maternal characteristics**			
Maternal age (year)	29.53 ± 6.2	28.86 ± 6.1	**0.008**
Parity	3.01 ± 2.2	2.78 ± 2.1	0.008*
Primipara	795 (31.8%)	344 (41.4%)	**< 0.001**
Gestation age at booking (week)	21.8 ± 8.8	22.2 ± 5.3	0.34
BMI (kg/m^2^)	29.45 ± 5.96	29.7 ± 6.04	0.23
GDM	354 (14.5%)	114 (14.3%)	0.85
Pre-existing diabetes:			
Type I	9 (0.37%)	6 (0.75%)	0.20
Type II	7 (0.28%)	5 (0.72%)	0 .20
Pregnancy induced hypertension	29 (1.2%)	11 (1.4%)	0.68
Preeclampsia	17 (0.7%)	5 (0.62%)	0.83
**Infant characteristics**			
Male, n (%)	1303 (53.6%)	412 (51.7%)	0.36
Gestational age (week)	39.12 ± 1.19	39.12 ± 1.195	0.85
Birth weight (kg)	3.2 ± 0.45	3.15 ± 0.46	0.012*
Birth length (cm)	49.89 ± 2.16	49.69 ± 2.27	**0.029**
Head circumference (cm)	34.26 ± 1.25	34.13 ± 1.21	**0.011**
Macrosomia (≥ 4.0 kg)	113 (4.64%)	30 (3.75%)	0.29
Low birth weight (<2.5 kg)	111 (4.56%)	41 (5.14%)	0.5

Mann–Whitney Test*.

Data expressed as either n (%) or mean ± standard deviation.

SHS = second-hand smoke, BMI = body mass index, GDM = gestational diabetes.

Bold figure indicates statistical significance.

The characteristics of infants of exposed and non-exposed women are shown in Table 1. Infants of women, who were exposed to SHS, had significantly less birth weight, were significantly shorter and their mean head circumference was significantly smaller than that of infants of unexposed mothers. The frequency of LBW infants (< 2.5 kg) was higher; however this difference did not reach statistical significance. Similarly, macrosomia was more frequent in non-exposed mothers but the difference was not statistically significant (Table 1).

Table 2 shows the anthropometric measurements of infants of women exposed and non-exposed to SHS stratified by BMI. The results showed significant trends of increased mean birth weight, mean length and mean head circumference measurements with the increase in maternal BMI. Similarly, there were significant trends of reduction in all of the above mentioned measurements of the newborn from mothers who shared the same BMI category and exposed to SHS compared to those who were not exposed (Table 2).

**Table 2:** The anthropometric measurements of infants of women exposed and non-exposed to SHS stratified by maternal BMI

**Anthropometric measurement**	**BMI**	**BMI**	**BMI**	**P value**
	**(<18)**	**(20–29.99)**	**(30+)**	
	**(n = 21)**	**(n = 1806)**	**(n = 1404)**	
**Mean birth weight (kg)**				
Exposed to SHS	2.67 ± 0.27	3.08 ± 0.49	3.12 ± 0.56	0.67
Non-Exposed SHS	3.04 ± 0.37	3.15 ± 0.44	3.27 ± 0.47	**<0.001**
**Mean length (cm)**				
Exposed to SHS	47.17 ± 1.94	49.45 ± 2.29	49.63 ± 2.56	**0.033**
Non-Exposed SHS	49.18 ± 1.88	49.77 ± 2.13	50.05 ± 2.18	**0.002**
**Mean HC (cm)**				
Exposed to SHS	32.83 ± 0.68	33.99 ± 1.22	34.22 ±1.22	**0.001**
Non-Exposed SHS	33.88 ±1.10	34.12 ±1.19	34.44 ± 1.28	**<0.001**

Data expressed as either n (%) or mean ± standard deviation.

SHS = second-hand smoke, BMI = body mass index, HC = head circumference.

Bold figure indicates statistical significance.

The odds of delivering a macrosomic baby increased by 9-fold for women with a BMI of ≥ 30 kg/m^2^ compared to non-obese women; OR 9.18, 95% Confidence Interval (CI) (1.01, 9.37), p = 0.04. However the risk of an obese mother delivering a macrosomic baby was attenuated to 1.5-fold in women exposed to SHS compared to those who were not exposed, OR 1.53, 95% CI (1.19, 12.1) (p <0.0001) (Table 3).

**Table 3:** Adjusted odds ratios for newborn macrosomia and low birth weight for women exposed and non-exposed to SHS stratified by BMI

**Newborn measurement**	**Non-obese**	**Underweight**	**Obese**
	**BMI = 18-29.99**	**BMI < 18**	**BMI ≥ 30**
	**(n = 1806)**	**(n = 21)**	**(n = 1404)**
**Macrosomia**			
Non-exposed to SHS			
Adjusted OR	1.0	0.57 (0.25, 1.29)	9.18 (1.01, 9.37)
		(p = 0.17)	**(p = 0.04)**
Exposed to SHS			
Adjusted OR	1.0	0.46 (0.31,1.78)	1.53 (1.19,12.1)
		(p = 0.68)	**(p < 0.0001)**
**Low birth weight**			
Non-exposed to SHS			
Adjusted OR	1.0	2.15 (1.001,4.57)	1.18 (0.26,15.9)
		**(p = 0.034)**	(p = 0.54)
Exposed to SHS			
Adjusted OR	1.0	2.71 (1.82,4.045)	2.15 (1.01,4.73)
		**(p = 0.02)**	**(p = 0.038)**

SHS = second-hand smoke, BMI = body mass index, OR = Odds ratio.

Bold figure indicates statistical significance.

The estimated risk of delivering a macrosomic baby was reduced to almost half in women who were underweight compared to those who were not obese, OR 0.57, 95% CI (0.25, 1.29) (p = 0.17) and was further attenuated for mothers exposed to SHS compared to those who were not exposed, OR 0.46, 95% CI (0.31, 1.78) (p = 0.68). However these values did not reach statistical significance (Table 3).

The odds of delivering an infant with birth weight <2.5 kg was more than doubled for women who were underweight compared to non-obese women, OR 2.15, 95% CI (1.001, 4.57), (p = 0.034), and were further increased to almost 3-fold for women who were exposed to SHS compared to non-obese unexposed women, OR 2.71, 95% CI (1.82, 4.045), (p = 0.02) (Table 3). The odds of delivering an infant with birth weight <2.5 kg were more in obese women compared to non-obese women, and were more than doubled in obese women who were exposed to SHS (Table 3).

## Discussion

The results of this study confirmed our previous findings and others’ about the effect of in-utero exposure to SHS on reducing the weight and other anthropometric measurements of the newborn [1,3,13]. In addition we demonstrated a trend of reduced newborn’s measurements with the incremental reduction in maternal BMI with further decrease in measurements in newborns of mothers exposed to SHS in the same BMI category. Furthermore we quantified the risk of macrosomia and LBW according to maternal BMI, with evident increased risk of macrosomia in obese women and increased risk of LBW in underweight women. It was evident from our results that there was an increased risk of LBW and decreased risk of macrosomia in women who were exposed to SHS in the same BMI category. These findings are consistent with the recent findings of Voigt et al. [7,14].

Although the effects of in-utero exposure to tobacco smoke and maternal obesity are expected to counteract each other with respect to LBW rate, they are nevertheless two pathological conditions with documented adverse effects on the newborn and the future adult [1,2,5,15]. In-utero exposure to nicotine and cotinine affects the development of the placenta and the balance of multiple vasoactive factors with subsequent reduction in oxygen delivery to the fetus [12,16]. On the other hand, the pathological effects of maternal obesity on the fetus are mediated through maternal adiposity and the associated circulating factors including inflammation mediators up-regulation, in addition to the increased insulin resistance associated with maternal obesity [17,18]. Due to the high prevalence of SHS and maternal obesity in this study, we believe that both conditions present major public health risks to the pregnant women and their infants.

Although the prevalence of maternal underweight in this study was only 0.65%, yet its adverse effects were significantly evident in the increased risk of the delivery of LBW infant and the decreased anthropometric measurements of infants born to this category of mothers compared to the other categories. These results are consistent with those reported by previous studies [19,20]. It was evident from our results that the combination of maternal underweight and exposure to SHS resulted in the worst possible outcomes with respect to the risk of delivering an LBW infant or an infant with the least anthropometric measurements compared to the normal weight mothers who were not exposed to SHS, which suggests a synergistic effect of maternal underweight and exposure to SHS. These results are in agreement with those recently reported by Voigt et al. [7,14].

We believe that the results of this study should direct reproductive health policy in the Kingdom of Saudi Arabia towards the implementation of programs aimed at smoke free environment for mothers and their children. In addition to the implementation of evidence based interventions for reducing and preventing obesity in pregnancy and its adverse effects on the mother and the fetus. It is important that women in reproductive age group are aware of the adverse consequences of obesity on the mother and her unborn child, which will motivate better utilization of effective interventions.

We are aware of the limitation of this study including the retrospective nature of the investigation and the lack of data on pre-pregnancy maternal weight due to the routine late booking for antenatal care in the hospital, considering that maternal adiposity correlates better with maternal pre-pregnancy and first trimester weight rather than maternal weight in late pregnancy [21]. However other measurements of maternal obesity such as gestational weight gain, central obesity and modified categories of BMI to define obesity during pregnancy, were found to be associated with adverse pregnancy outcomes such as macrosomia and increased risk of caesarean section [22-24]. Another limitation of this study is that the exposure to SHS was based on the women’s self-reporting without the use of a biomarker to verify exposure. This might have skewed our results, considering the high rate of misclassification reported for both active and SHS exposure in pregnancy [25,26]; however such misclassification was not detected, when self-reported SHS was compared to biomarker detected SHS, in some communities in the Middle East [27]. Additionally we did not quantify the exposure to SHS by the number of hours; hence we did not report a dose response relationship between exposure to SHS and pregnancy outcomes; nevertheless due to the self-reporting design of the study and the possibility of recall bias, a dose response might not have been verified.

## Conclusion

Exposure of Saudi pregnant women to SHS is associated with reduced birth weight, head circumference and shorter length of the newborn as well as increased rate of LBW infants, irrespective of maternal BMI. Maternal obesity is associated with increased risk of delivering a macrosomic infant; conversely maternal underweight is associated with the increased risk of delivering an LBW infant.

## Competing interests

The authors would like to declare that they have no conflict of interest with the contents of this article.

## Authors’ contributions

HW conceived the idea of the study, and was responsible for writing the final study manuscript. AM participated in the statistical analysis and write-up of the article. AB conducted the statistical analysis and participated in the write-up of the article. RZ, participated in the write-up of the article and AF participated in the statistical analysis and results interpretation. All authors reviewed and approved the final manuscript.

## Pre-publication history

The pre-publication history for this paper can be accessed here:

http://www.biomedcentral.com/1471-2458/13/1058/prepub
